# The Influence of DMSO on PVA/PVDF Hydrogel Properties: From Materials to Sensors Applications

**DOI:** 10.3390/gels11020133

**Published:** 2025-02-13

**Authors:** Giada D’Altri, Angelica Giovagnoli, Valentina Di Matteo, Lamyea Yeasmin, Stefano Scurti, Isacco Gualandi, Maria Cristina Cassani, Silvia Panzavolta, Mariangela Rea, Daniele Caretti, Barbara Ballarin

**Affiliations:** 1 Department of Industrial Chemistry “Toso Montanari”, Bologna University, Via Piero Gobetti 85, I-40129 Bologna, Italy; angelica.giovagnoli2@unibo.it (A.G.); valentina.dimatteo5@unibo.it (V.D.M.); lamyea.yeasmin@unibo.it (L.Y.); stefano.scurti2@unibo.it (S.S.); isacco.gualandi2@unibo.it (I.G.); maria.cassani@unibo.it (M.C.C.); daniele.caretti@unibo.it (D.C.); 2 Politecnico di Torino, Corso Duca degli Abruzzi, 24-10129 Torino, Italy; 3 Center for Industrial Research-Advanced Applications, Mechanical Engineering and Materials Technology CIRI MAM, University of Bologna, Viale del Risorgimento 2, I-40136 Bologna, Italy; 4 Department of Chemistry “Giacomo Ciamician”, University of Bologna, Via Piero Gobetti 83, I-40129 Bologna, Italy; silvia.panzavolta@unibo.it (S.P.); mariangela.rea2@unibo.it (M.R.); 5 Center for Industrial Research-Fonti Rinnovabili, Ambiente, Mare e Energia CIRI FRAME, University of Bologna, Viale del Risorgimento 2, I-40136 Bologna, Italy

**Keywords:** PVA/PVDF hydrogel, flexible sensors, flexible electronics, DMSO

## Abstract

This research study aims to explore the synergistic effects of incorporating polyvinylidene fluoride (PVDF) into polyvinyl alcohol (PVA) hydrogels to enhance their suitability for triboelectric sensors applications. The preparation process employs a method of freezing/thawing conducted in dimethyl sulfoxide (DMSO), followed by solvent replacement with water. This approach effectively preserves PVDF in its α phase, eliminating piezoelectric effects and enhancing the hydrogels’ mechanical properties. The use of DMSO contributes to reduced pore size, while incorporating PVDF significantly improves the three-dimensional network structure of the hydrogels, resulting in enhanced thermal and chemical resistance. Thorough characterization of the resulting PVA/PVDF composite hydrogels, prepared with varying ratios of PVA to PVDF (10:0, 8:2, and 5:5), was conducted by using scanning electron microscopy (SEM), Fourier transform infrared spectroscopy (FTIR), electrochemical impedance spectroscopy (EIS), rheology, and thermogravimetric analysis (TGA). Notably, the composite hydrogels were tested in pressure sensors and human voice sensors, demonstrating their capability to recognize different patterns associated with various letters. The incorporation of PVDF significantly enhanced the signal-to-noise ratio in PVA/PVDF-based sensors compared with those made solely from PVA, highlighting a notable improvement in voice detection. The enhancements were quantified as 56% for “a”, 35% for “r”, and 47% for “m”.

## 1. Introduction

Hydrogels prepared by using water and dimethyl sulfoxide (DMSO) as dispersant media have garnered significant attention due to their unique properties and versatility in various applications [1,2,3]. The use of DMSO in hydrogels plays a critical role in polymeric network formation, due to the strong capacity to interact with water molecules, thereby influencing the hydrogen bonding network of hydrogels. DMSO can strengthen or weaken the hydrogen bonds between polymer chains, depending on solvent composition and concentration [4,5]. Increasing DMSO concentration generally results in decreased swelling due to enhanced intra- and intermolecular hydrogen bonding within the polymer network [5] and increased stiffness and strength of the hydrogel [6,7]. For instance, hydrogels prepared with a higher proportion of DMSO show marked improvements in elasticity and tensile strength compared with those made with pure water. Studies have shown that hydrogels prepared with DMSO exhibit a 9–12-fold increase in storage and loss moduli (G′ and G′′) compared with those without DMSO [5]. The use of DMSO enhances the optical transparency of hydrogels, particularly in poly(vinyl alcohol) (PVA) hydrogels [8]. Higher concentrations of DMSO lead to lower equilibrium swelling, resulting in a more homogeneous structure that allows for better light transmission [9]. DMSO influences the pore size of hydrogels, resulting in smaller pores compared with those prepared in pure water. This fine control over porosity can enhance the performance of hydrogels in applications such as drug delivery and tissue engineering [10]. Moreover, DMSO enhances the anti-freezing properties of hydrogels, making them suitable for applications in cold environments or where temperature fluctuations are expected [11] and some DMSO-based hydrogels exhibit thixotropic behavior, allowing them to transition rapidly between gel and quasi-liquid states and vice versa, which enhances their usability in dynamic environments [5]. Finally, mixing DMSO with other solvents, such as water, allows for flexibility and versatility in formulating composite hydrogels with varying properties by adjusting concentrations and ratios according to the desired characteristics [10].

Among the composite hydrogels, polyvinyl alcohol (PVA) and polyvinylidene fluoride (PVDF) (PVA/PVDF) composite hydrogels represent a promising class of materials with significant potential across various applications due to their enhanced mechanical properties, biocompatibility, and possible piezoelectric performance [3,12,13]. Their ability to combine the strengths of both polymers opens new avenues for innovation in fields ranging from healthcare to biomedical engineering [14] and environmental remediation [15].

Polyvinylidene fluoride (PVDF) is a highly versatile thermoplastic fluoropolymer known for its exceptional properties [14,16], which make it suitable for a wide range of applications. PVDF exhibits excellent resistance to a variety of chemicals, including acids and solvents, making it suitable for harsh environments; it has high thermal stability due to the high melting point (up to 150 °C) and maintains its properties at elevated temperatures, which is beneficial for high-temperature applications [16]. PVDF is an effective electrical insulator, often used in electrical and electronic applications due to its low dielectric constant, and possesses good mechanical strength and toughness, allowing it to withstand significant mechanical stresses [16]. Moreover, PVDF exists in five crystalline phases: α, β, γ, δ, and ε [14,17]. Among these, the β phase is particularly significant due to its strong piezoelectric properties, which arise from the orientation of dipoles within the polymer’s structure [16,18]. Annealing PVDF at around 120 °C promotes this transformation [14,18].

Polyvinyl alcohol (PVA) hydrogels are widely studied for their unique properties and versatile applications, particularly in the biomedical field [13,19] due to their excellent biocompatibility, making them suitable for use in wound dressings, drug delivery systems, and tissue engineering [19]. These hydrogels exhibit high mechanical strength and elasticity, and they can retain a significant amount of water, often exceeding 90% by weight, which contributes to their flexibility and makes them effective in applications requiring moisture retention [20]. Both polymers are currently implemented in smart devices that have piqued researchers’ interest due to a major focus on Human–Machine Interface (HMI) systems [21]. As technologies evolve through more biocompatible and bio-inspired devices, the need for flexibility, simplicity, and versatility in smart devices arises. Devices based on smart hydrogels have been implemented in heart [22] or breath monitoring [23] while also paving the path for biocompatible, lightweight, and flexible devices for voice sensing and word recognition [24,25,26]. Such results were obtained by exploiting capacitive, piezoelectric, piezoresistive, and triboelectric effects.

Different from what is reported in the literature, where reference is made to the piezoelectric properties of PVDF, in this paper, we investigate how the addition of PVDF can influence the properties of a PVA-based hydrogel obtained in DMSO. The PVDF was maintained in its α phase, thus excluding the influence of a possible piezoelectric effect. A distinctive preparation process involving freezing/thawing and solvent replacement was optimized to prepare PVA/PVDF composite hydrogels [5,14]. The hydrogels were characterized in depth, structurally and electrochemically. Finally, these composite hydrogels were tested as smart materials for pressure and human motion and voice sensor preparation. The results obtained indicate that this composite hydrogel is a valid alternative to the materials employed in wearable sensors like bioactive glasses [27], polydimethylsiloxane/carbon nanotube (PDMS/CNT) composites [28], PVDF films [29], piezoelectric hydrogels (PHs) [30], and conductive polymers [31,32]. The advantages of the proposed hydrogel are easier processability into various forms, including films, coatings, and three-dimensional structures, allowing for tailored designs in sensors applications; the versatility of application in various sensing modalities; and comparable or higher biocompatibility. To our knowledge, no similar investigation has yet been reported in the literature.

## 2. Results and Discussion

### 2.1. Hydrogel Characterization

**Figure 1:**
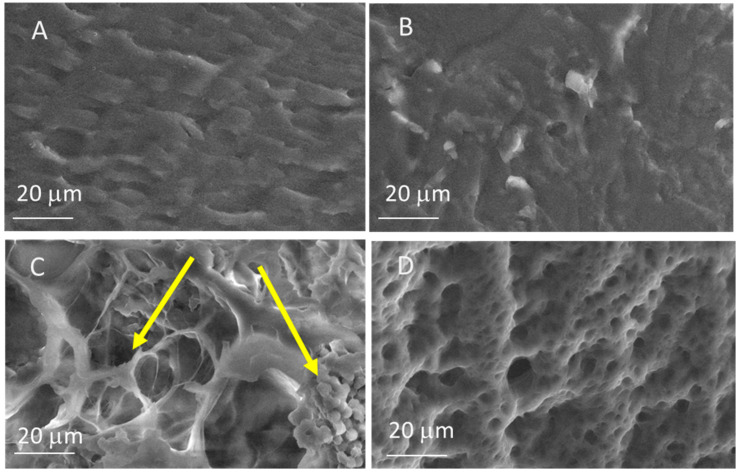
SEM images of PVA/PVDF hydrogel samples with different compositions. (**A**) 10:0; (**B**) 8:2; (**C**) 5:5; (**D**) PVA hydrogel prepared in an acidic aqueous media. In (**C**) the phase separation between PVA and PVDF is visible, as highlighted by the yellow arrows.

Due to difficult gelation in DMSO, we used a modified freezing/thawing (F/T) cycle combined with a solvent replacement method to prepare the hydrogel [5,14,33]. Three F/T cycles were carried out for 24 h instead of 16 h at −18°. Hydrogel morphology was investigated by SEM microscopy, and the related images of hydrogels with different PVA/PVDF weight ratios (5:5, 8:2, and 10:0) are reported in Figure 1A–C. Moreover, they were compared with the PVA hydrogel prepared in an acidic aqueous solution (1.0 M H_2_SO_4_) (Figure 1D).

The presence of DMSO during the gelation process leads to a denser hydrogel structure with reduced porosity as observable from the SEM image in Figure 1A. In fact, by comparing the morphology of the hydrogels prepared in pure DMSO (3A) and in the H_2_SO_4_ aqueous solution (3D) a significant change in porous distribution and network structures can be observed [9,10,34]. This result highlights the role of DMSO, which alters the gelation mechanism and contributes to a more homogeneous gel structure, leading to the formation of gels with different structures and properties [9]. The presence of separated regions due to the addition of PVDF in the 8:2 weight ratio was not observed; thus, the sample presented a similar morphology (Figure 1B). Only in the 5:5 PVA:PVDF weight ratio, we observed an increase in porosity with a structure comparable to the PVA hydrogel sample prepared in aqueous media (Figure 1C). The images reveal the coexistence of two distinct phases with differing morphologies, indicating incomplete compatibility between the polymers in the blend or an antagonistic effect of PVDF during the cryo-structuration process, which hinders the full development of the open porous morphology [35].

**Figure 2:**
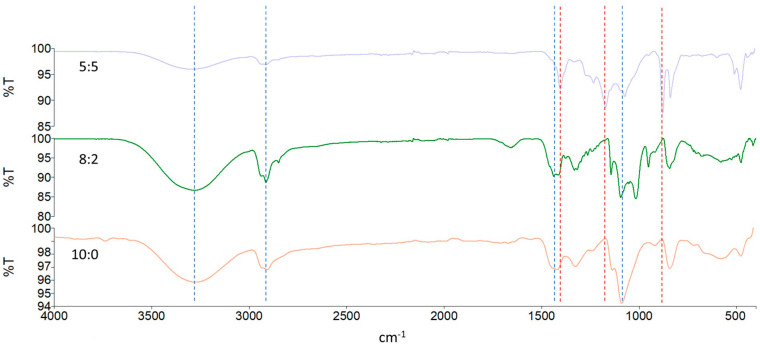
ATR-FTIR spectra of 5:5, 8:2, and 10:0 PVA/PVDF hydrogels. PVA typical peaks are marked by the dashed line in blue, and α-phase PVDF ones are marked by the dash line in red.

In all the spectra reported in Figure 2, the broadband characteristic of the intermolecular bonds of the hydroxyl groups is observed at 3500–3000 cm^−1^, due to OH stretching in the PVA macromolecules. The double peak that occurs at 2940 cm^−1^ is caused by the C-H stretching of the alkyl chains. The two peaks at 1414 and 1326 cm^−1^ are attributed to the coupling of the O-H interplane bending and the C-H wagging vibrations. The signal at 1085 cm^−1^ is assigned to the stretching vibration of C-O [36].

Information on the presence and structural characteristics of PVDF comes from the IR region between 450 and 1400 cm^−1^ [37,38]. The α phase presents typical bands at 1400 cm^−1^ (C–H bending), 1180 cm^−1^ (C–F stretching), 976 cm^−1^ (C-H twisting), 873 cm^−1^ (C–H wagging), and 830 cm^−1^ (C–F bending). Other bands at 490, 613, and 762 cm^−1^ attributable to CH_2_ bending confirm the α phase.

**Figure 3:**
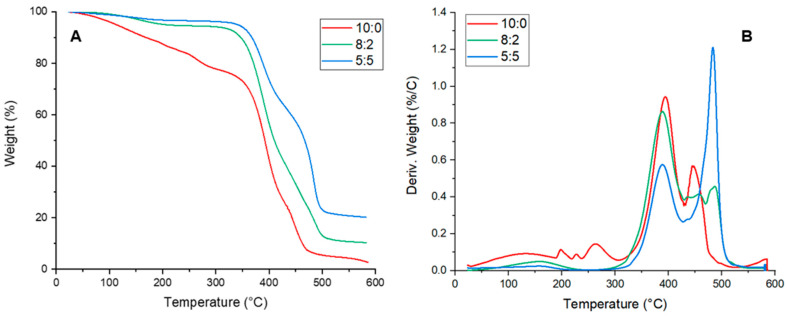
(**A**) TGA and (**B**) dTGA of 10:0, 8:2, and 5:5 PVA/PVDF samples.

The thermal stability of the samples was evaluated by TGA analysis, as reported in Figure 3A,B. All the thermograms exhibited several degradation steps: There was an initial weight loss at around 130 °C, due to the simultaneous solvent evaporation of water and DMSO mixture from the hydrogel matrix. The second degradation step between 350 and 450 °C, common to all samples, represented the thermal decomposition of PVA. In the 8:2 and 5:5 samples, a further step around 500 °C could be observed corresponding to the breaking of C-F bonds of PVDF. Moreover, by increasing the amount of PVDF in the polymer blend, the weight loss related to this degradative phase became more pronounced, as observed in the derivative TGA graph (Figure 3B). Concerning the residual portion, it increased with PVDF weight until it reached 20% in the 5:5 samples.

#### 2.1.1. Rheological Characterization

**Figure 4:**
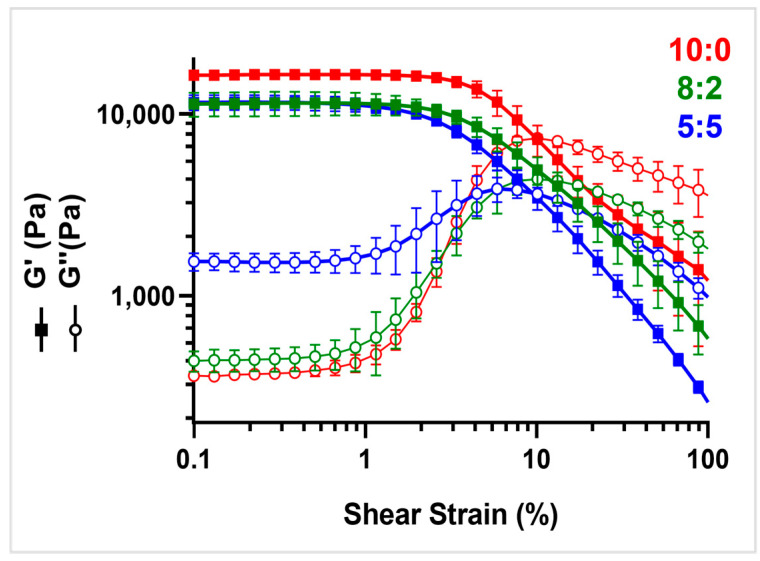
Amplitude sweep analysis of PVA/PVDF hydrogels.

The three different PVA-PVDF hydrogels were subjected to rheological analysis to highlight the influence of PVDF on the crosslinking degree and rigidity of the samples. Figure 4 reports the amplitude sweep curves, where all samples are characterized by G′ > G″, showing a gel-like behavior. Table 1 reports G′ and G″ values, as well as the LVER and cross-over points, which provide insights into the elasticity of the sample and breaking points, respectively, where interactions are broken and macromolecular chains start flowing. Also, the degree of crosslinking is reported, calculated as the height of the G″ peak of each curve. It can be noticed that the 10:0 samples, i.e., plain PVA, are characterized by the highest G′ and by the highest difference between G′ and G″ (red curves in Figure 4), underlining the strong crosslinking of the resulting network. By increasing the amount of PVDF in the hydrogel mixture, the crosslinking degree decreases, as can be noticed by the progressively lower distance between the G′ and G″ curves and by the obtained results of the crosslinking degree reported in Table 1. Also, the LVER is progressively reduced by increasing the PVDF amount. The 5:5 sample (blue curves in Figure 4) is the softest and easier to break. Even if the crosslinking degree is much reduced with PVDF introduction, the materials still show a high storage modulus and a cross-over point very similar to the 10:0 one. In conclusion, it can be stated that increasing the amount of PVDF in the hydrogel mixture decreases the crosslinking degree, without significantly affecting the materials’ resistance to flow. Therefore, by changing the PVDF amount, it is possible to tune the resulting materials’ softness.

#### 2.1.2. Electrochemical Characterization

The electrochemical impedance measurements are reported as a Nyquist plot (Figure S1). The graphs show a typical trend of an electrochemical circuit that has semicircle curves which does not close completely on the *x*-axis, suggesting the presence of resistive components in the system, a charge transfer process limited by the charge transfer resistance (Rct), and double-layer capacitance (Cdl) [39]. Resistive values of 7600 Ω and 4900 Ω were obtained for the 5:5 and 8:2 hydrogels, respectively.

### 2.2. Hydrogel Sensors Application

#### 2.2.1. Pressure Sensor

The PVA/PVDF hydrogels were used as active materials for the fabrication of pressure sensors. The gel was cut into a parallelepiped shape and encapsulated in food-grade silicone. Sensor production was completed by inserting a platinum wire into the encapsulation system which was in contact with the gel and the external environment. The measurement was performed in single-electrode mode [40], recording the short-circuit current (I) flowing between the Pt wire and the ground as a function of time. The aqueous phase within the gel, which has high resistance, can be viewed as constant load resistance that is usually present in the one-electrode measure set-up. The response of the PVA/PVDF hydrogel sensors was conducted by monitoring the current response as a function of time while the sensor was being perturbed with pressure generated from standard weights with masses of 1, 2, and 5 g placed on and lifted manually from the sensor surface. Thirty perturbation cycles consisting of weight loading lasting 5 s/weight unloading at intervals of 10 s were used for each weight.

**Figure 5:**
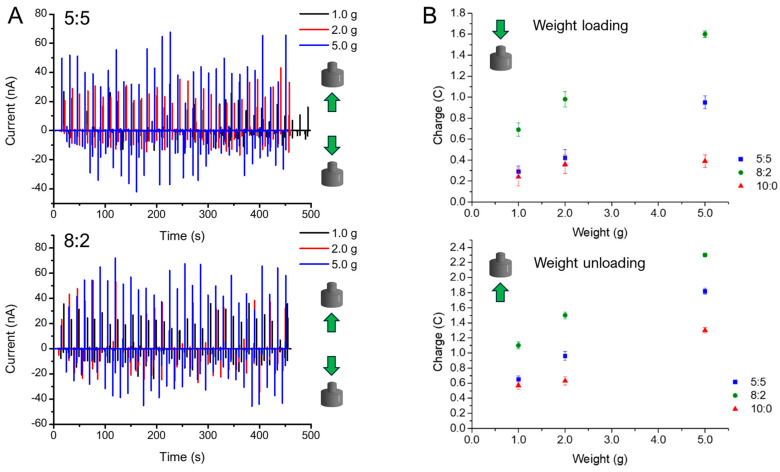
(**A**) Current as a function of time for different weights applied on 8:2 and 5:5 sensors. (**B**) Charge-versus-weight linear fits for 10:0, 8:2, and 5:5 sensors (loading and unloading results). The investigated weight motion towards the sensor is shown through green arrows.

Figure 5A illustrates the current trend as a function of time for different weights applied to 5:5 and 8:2 sensors. When weights are applied to the sensor, the current (I) rapidly reaches relatively high negative values. Subsequently, the current returns to its initial value, resulting in an overall response with a peak in the negative direction. Conversely, when the weight is removed from the sensor, a current peak in the positive direction is recorded. Notably, the charges observed during the unloading phase are consistently greater than those recorded during the loading phase. It can be seen how the variation in the intensity of the current follows the variation in the applied pressure: smaller pressure produces weaker current signals (black curves), while greater pressure generates more intense signals (blue curves).

The curves depicting the short-circuit current (I) as a function of time were analyzed to extract the peak current and short-circuit charge (C) values for both the loading and unloading phases. The charge was calculated by integrating the current obtained from the short-circuit curves over time. The 30 peak current and charge values were averaged to derive the mean value and the corresponding standard deviation in all experiments. The signals extrapolated as charge exhibited a percentage standard deviation ranging from 3% to 10%, while the data extrapolated as peak current demonstrated a percentage standard deviation between 21% and 60%. These results indicate that peak charge facilitates the assessment of pressure variation with lower uncertainty; consequently, it was employed in subsequent analyses.

Figure 5B reports the charge vs. weight applied to 5:5 and 8:2 sensors compared with the 10:0 one, where PVDF is absent. No linear trend was observed in the 10:0 sample, where the PVDF presence in α-phase increased the response signal with a linear response, while the charge was linearly dependent on the weight for the sensor prepared with PVA/PVDF hydrogel, highlighting the key role of PVDF in the sensing mechanism. The results of the linear fitting are reported in Table 2. As expected from the current curves, the slope obtained during unloading is always greater than that recorded during loading. Moreover, the sensor prepared with the 8:2 PVA/PVDF composition exhibits a higher response than the devices made of 5:5 PVA/PVDF. The superior performance of the 8:2 PVA/PVDF hydrogel-based device can be ascribed to the greater homogeneity of the microstructure observed in SEM, which does not exhibit the segregation of different polymeric phases that is present in the 5:5 PVA/PVDF hydrogel.

The device operating principle has been hypothesized by considering the characterization of the material and the recent literature. PVDF plays a key role, as demonstrated by the unsatisfactory performance of the device made solely with PVA hydrogel. However, piezoelectric transduction is excluded, since the characterization of the proposed hydrogels shows only the formation of the α phase of PVDF, which, having an overall dipole moment equal to zero, does not exhibit piezoelectric behavior. Similarly to what was proposed by Abir et al. [41] for a pressure sensor made from a TPU and PVDF-based composite material, transduction may be attributed to triboelectric effects, whereby the electronegative PVDF withdraws electrons from the other components of the hydrogel. The application of a periodic mechanical stimulus, similar to the calibration procedure used, allows for the formation of static charge on the material, enhancing the sensor’s performance [42,43].

#### 2.2.2. Human Voice Sensor

**Figure 6:**
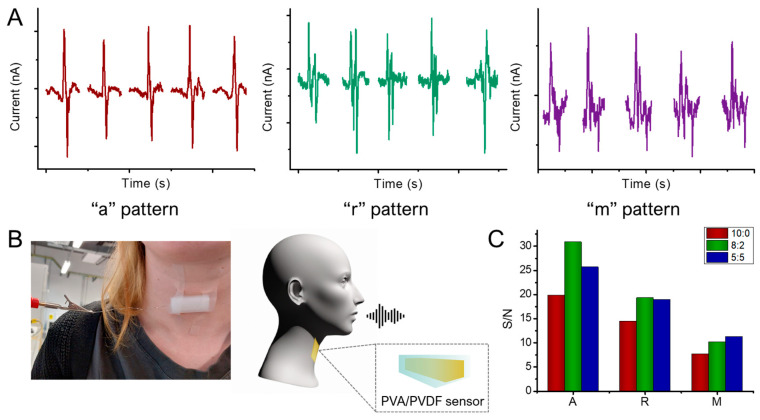
(**A**) Patterns related to pronunciation of different letters in chronoamperometry measurements. (**B**) Experimental set-up with hydrogel sensor attached to a volunteer’s throat and schematic process of device functioning. (**C**) Signal-to-noise ratio for pronunciation of single selected letters with each device.

The fabricated devices were employed for voice sensing, by exploiting the throat mechanical vibration during the speaking phase [44]. To test the possible application, the hydrogel sensor was attached to a volunteer’s throat and connected through a Pt wire to the potentiostat in the one-electrode set-up previously implemented (Figure 6B). The measurements were conducted by clearly pronouncing single selected letters, A, R, and M, at intervals of 30 s for five times. The speaking phase thus produces a mechanical vibration in the throat, which induces a possible triboelectric effect between the polymeric regions of the sensor, with consequential electron transfer from the PVA region to the PVDF one [40]. To further compare the devices, the signal-to-noise ratio (S/N) was calculated by the average of the maximum values of the peaks in each measurement (Figure S2) with respect to the standard deviation of the blank. The results highlight the performance differences between the PVA/PVDF blend devices and the PVA one, as presented in Figure 6C. As shown, the letter “A” provides the most significant S/N ratio, due to vocal vibration. On the other hand, the letter “M” does not allow optimum signal recognition, caused by the less intense vibration produced by pronunciation. The devices made from 8:2 PVA/PVDF and 5:5 PVA/PVDF hydrogels show better performance than the devices obtained with only PVA. Although 10:0 PVA/PVDF can detect the voice-generated signal, the signal-to-noise ratio is very low for “M”, the most challenging letter, highlighting the advantage due to the presence of PVDF. Moreover, observing the registered current signals, the different patterns of the selected letters can be confirmed by the recurring patterns presented for each letter shown in Figure 6A. The presence of a distinctive signal related to specific letters can open the doors to future research in terms of word recognition and the implementation of artificial intelligence in the process.

As shown in Table S2, other recent advanced materials present flexibility, mechanical properties, and sensitivity while maintaining interesting performance, but they often require complex fabrication and preparation with respect to the PVA/PVDF hydrogels present in this work. Moreover, the simplicity and versatility of the presented design allow for a wider range of different fields of applications.

## 3. Conclusions

The present study explored in detail the preparation and properties of polyvinyl alcohol (PVA)- and polyvinylidene fluoride (PVDF)-based hydrogels by using a freezing/thawing method combined with solvent replacement. The results obtained offer important insights into the optimization of the mechanical and functional properties of these polymeric materials.

The use of DMSO resulted in a denser hydrogel structure with reduced pore size, highlighting how the solvent influences the gelation mechanism. The presence of DMSO contributed to greater homogeneity in the gel structure, eliminating highly porous and locally oriented regions.

Morphological analyses revealed that the proportions of PVA/PVDF significantly influence the morphology of the hydrogels. The 5:5 PVA:PVDF sample showed increased porosity but exhibited two distinct phases, likely due to polymer incompatibility or the inhibitory role of PVDF in forming a fully open porous morphology. Instead, thermal analysis showed that the co-presence of the PVDF in the hydrogel increases the thermal stability of the material compared with the PVA-based sample. In this way, it is possible to modulate the properties of hydrogels by varying the PVA/PVDF ratio, offering opportunities to design materials with specific characteristics for targeted applications. Electrochemical measurements revealed a promising application of these hydrogels as pressure sensors, as well as an interesting voice sensor application. Specifically, the device was tested for single-letter pronunciation, providing a notable S/N ratio for PVA/PVDF blend devices and letter recognition through the observable patterns in chronoamperometry measurements.

In summary, this study demonstrated that PVA/PVDF hydrogels prepared in DMSO exhibit improved physical and rheological properties, making them suitable for advanced applications in the fields of sensing and materials engineering compared with the other systems present in the literature. Furthermore, the easy preparation methodology and the possible scalability of the process make this material suitable for sensing applications. The findings obtained provide a solid basis for further research and development, suggesting that the optimization of polymer formulations and preparation conditions can lead to significant innovations in the field of smart materials.

## 4. Materials and Methods

### 4.1. Materials

Polyvinyl alcohol (PVA; Mw: 89,000–98,000 (+99% degree of hydrolysis); Sigma Aldrich, Merck KGaA, Darmstadt, Germania), polyvinylidene fluoride (PVDF; 95.0–98% H_2_SO_4_), dimethyl sulfoxide (DMSO), and ACS reagent (>99.9%; Sigma Aldrich, Merck KGaA, Darmstadt, Germania) were used. Two-component silicone rubber for food molds (Reschimica, Barberino Tavarnelle (FI), Italy) was employed for template material preparation.

### 4.2. PVA/PVDF Hydrogel Preparation

We investigated three different hydrogel compositions with varying PVA:PVDF *w*/*w* ratio: 10:0, in which only PVA was employed; 8:2; and 5:5. First, the selected amount of PVA and PVDF necessary for the desired *w*/*w* ratio was weighed and dissolved in two flasks with DMSO at 70 °C under stirring (Table S1). Then, the two solutions were mixed, homogenized, and transferred into a Petri dish.

To prepare the hydrogel, the freezing/thawing method combined with solvent replacement was used [5,33]. The formation of hydrogels typically involves physical cross-linking through hydrogen bonding between hydroxyl groups in PVA, which increases the structural integrity of the hydrogel. This method enhances hydrogen bonding between PVA polymeric chains, promoting the formation of crystalline domains in the final network [45,46]. By changing a protocol reported in the literature [14,33], we used three phases: (1) physical cross-linking obtained through three freezing/thawing (F/T) cycles (−18 °C for 24 h), (2) replacement of the organic solvent with water to obtain hydrogel (obtained by immersion in distillate water for 2 days, replacing the water every 12 h), and (3) a further three freezing/thawing cycles (−18 °C for 3 h). After the F/T cycles, the final hydrogel is pale brown.

For the hydrogel obtained in acidic aqueous media, 1.5 g of PVA powder was dissolved in 15 mL of aqueous 1.0 M H_2_SO_4_ solution (PVA/H_2_SO_4_ weight-to-volume ratio equal to 1/10) at 70–90 °C under vigorous stirring to obtain a transparent solution. The solution was then ultrasonically treated to remove bubbles and poured into a Petri dish. Three freeze-thaw cycles at −18 °C for 3 h were used to obtain a hydrogel membrane [18].

### 4.3. Hydrogel Sensor Assembling

**Figure 7:**
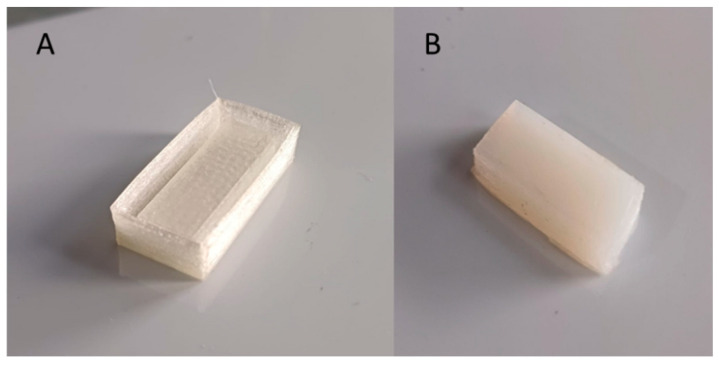
(**A**) TPU mold; (**B**) silicon rubber encapsulated hydrogel sensor.

The hydrogels were encapsulated in a food-grade silicone rubber sheath to prepare the sensor. Commercial two-component silicone rubber consisting of two components (A, a silicone base, and B, a catalyst or hardener) that need to be mixed for a few minutes before use to activate the curing process was used. An A:B ratio of 2:3 was used. The rubber was then placed in a thermoplastic polyurethane (TPU) mold measuring 3.0 cm × 1.0 cm, previously printed with a 3D printer, and left to solidify for at least 3 h. A portion of hydrogel was cut to the exact size of the silicone sheath and inserted inside. More silicone rubber was poured on top and left to solidify as in Figure 7.

### 4.4. Characterization

#### 4.4.1. Morphological, Rheological, and Chemical-Physical Characterization

The morphology of the hydrogel samples was observed with a scanning electron microscopy (SEM) Renishaw field-emission scanning electron microscope, equipped with an InLens detector, operating at 10 kV and a current of 80 pA. Before analysis, the samples were lyophilized.

The ATR-FTIR spectra were collected by using a Perkin Elmer Spectrum Two spectrometer (710 Bridgeport Avenue Shelton, CT 06484-4794, United States), equipped with a Universal ATR accessory, with a resolution of 0.5 cm^−1^ in the range 4000–400 cm^−1^ using 40 scans.

Thermogravimetric analysis was conducted with a NETZSCH TG 209F1 Libra thermogravimetric analyzer (Verona, Italy) in an inert atmosphere under N_2_ with a heating ramp from 25 °C to 600 °C and a heating rate of 20 °C/min.

The rheological properties of the PVA-PVDF hydrogels were carried out by an MCR 102 parallel-plate rheometer (Anton Paar, Graz, Austria) in a plate–plate geometry with a diameter of 25 mm (PP-25 plate) and a gap of 2.3 ± 0.2 mm. The samples were deposited onto the rheometer plate with a spatula. Subsequently, the upper plate was lowered until it came into contact with the sample surface. The excess material was removed, and the trap was filled with distilled water to avoid evaporation phenomena. An oscillatory amplitude sweep test was carried out to assess the storage (G′) and loss (G″) moduli, the cross-over point (G′ = G″), and the linear viscoelastic range (LVER) at 25 °C in a shear strain amplitude range from 0.01 to 100%, with a fixed frequency value equal to 5 rad/s. Results are reported as means ± SDs, with *n* = 3.

#### 4.4.2. Electrochemical Test

Electrochemical Impedance Spectrometry (EIS) measurements were performed by using an Autolab GSTAT128 N potentiostat/galvanostat (Metrohm-Autolab, Origgio (VA) Italy) controlled by NOVA 2.10 software. A Swagelok-type cell with 316 stainless steel electrodes of 1.0 cm in diameter, connected to the working electrode and the reference electrode at the two poles, was used (Figure S3). EIS measurements were conducted at room temperature, with an alternating voltage amplitude of 10 mV and in a frequency range of 0.01 to 10^5^ Hz.

**Figure 8:**
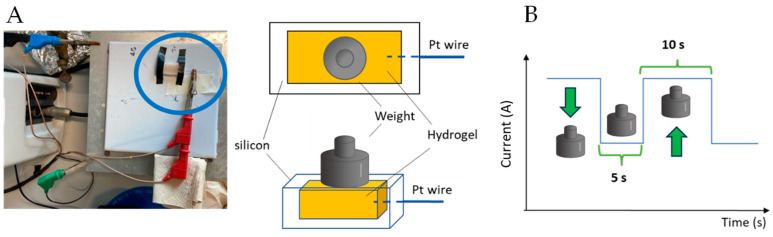
(**A**) Experimental set-up and (**B**) descriptive scheme for the chronoamperometric measures.

Chronoamperometric measurements were performed with the same Autolab potentiostat/galvanostat used for the EIS test. The connection between the potentiostat and the sensor was made through a platinum wire inserted into the hydrogel. The measurements were carried out by manually placing different standard weights (1, 2, or 5 g) on the sensor surface, waiting for 5 s, and removing them for 10 s to allow the system to stabilize. For each weight, 30 measurements were made. The experimental set-up appears in Figure 8.

## Figures and Tables

**Table 1:** G′ and G″ values, LVER%, and Cross-over point (%) of PVA: PVDF samples with different ratios.

PVA:PVDF	G′ (kPa)	G″ (kPa)	LVER (%)	Cross-Over (%)	Crosslinking Degree
5:5	11 ± 1	1.5 ± 0.2	0.7%	9%	2304
8:2	11 ± 2	0.45 ± 0.04	0.8%	12%	3908
10:0	16.5 ± 0.4	0.37 ± 0.02	1.5%	10%	6733

**Table 2:** Linear fit parameters.

Sensor	8:2	5:5	8:2	5:5
	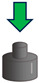 Weight Loading		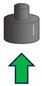 Weight Unloading	
Slope	0.22	0.166	0.29	0.294
Error	0.01	0.008	0.09	0.004
Intercept	0.49	0.11	0.86	0.36
Error	0.05	0.03	0.03	0.01
R^2^	0.999	0.999	0.995	0.999

## Data Availability

The original contributions presented in this study are included in the article/Supplementary Material. Further inquiries can be directed to the corresponding authors.

## Supplementary Materials

The following supporting information can be downloaded at: https://www.mdpi.com/article/10.3390/gels11020133/s1, Figure S1: Nyquist plot obtained with 8:2 and 5:5 hydrogels, Figure S2: Comparison of current vs. time signal for letters “a”, “r”, and “m”, obtained with 5:5, 8:2, and 10:0 sensors, Figure S3. Experimental set-up of the Swagelok cell (left) and scheme of the electrode and hydrogel assembly (right), Table S1: Reports the preparative conditions used to prepare the different PVA/PVDF based hydrogel in DMSO solvent, Table S2: Recent advanced materials used in sensors applications [47,48,49,50,51,52,53].
