# Supplementary material for: The Influence of DMSO on PVA/PVDF Hydrogel Properties: From Materials to Sensors Applications
Source: Gels. 2025 Feb 13;11(2):133. doi: 10.3390/gels11020133 (PMC11854958; doi:10.3390/gels11020133)
Supplement: Supplementary file 1 [file gels-11-00133-s001.zip › gels-3458024-supplementary.pdf]

The influence of DMSO on PVA/PVDF Hydrogel Properties: From materials to sensors applications

Giada D'Altri[a*], Angelica Giovagnoli[a], Valentina Di Matteo[a], Lamyea Yeasmin[a,b], Stefano Scurti[a], Isacco Gualandi[a,c], Maria Cristina Cassani[a,c], Silvia Panzavolta[d,] Mariangela Rea[d], Daniele Caretti[a] and Barbara Ballarin,[a,c,e*]

[a]Department of Industrial Chemistry "Toso Montanari", Bologna University, Via Piero Gobetti 85, I-40129, Bologna, Italy. *UdR INSTM of Bologna*

[b]Politecnico di Torino, Corso Duca degli Abruzzi, 24, I-10129 Torino, Italy

[c]Center for Industrial Research-Advanced Applications in Mechanical Engineering and Materials Technology CIRI MAM University of Bologna, Viale del Risorgimento 2, I-40136 Bologna, Italy.

[d]Department of Chemistry "Giacomo Ciamician", University of Bologna, Via Piero Gobetti 83, I-40129, Bologna, Italy.

[e]Center for Industrial Research-Fonti Rinnovabili, Ambiente, Mare e Energia CIRI FRAME University of Bologna, Viale del Risorgimento 2, I-40136 Bologna, Italy.

*To whom correspondence should be addressed. E-mail: barbara.ballarin@unibo.it (B.B), tel: +39 051 2093700; giada.daltri2@unibo.it (G.D.).

Supporting Information

**Hydrogel Preparation**	**2**
**Electrochemical Characterization**	**3**

Hydrogel Preparation

Table S1 reports the preparative conditions used to prepare the different PVA/PVDF based hydrogel in DMSO solvent

Table S1. PVA/PVDF hydrogel preparative conditions

**PVA/PVDF**	**5:5**	**8:2**	**10:0**
**PVA**	0.917 g	1.467 g	1.833 g
**PVDF**	0.917 g	0.367 g	0 g
**DMSO for PVA**	7.5 mL	12.0 mL	15.0 mL
**DMSO for PVDF**	7.5 mL	3.0 mL	0 mL

Electrochemical Characterization

Electrochemical Impedance spectrometry measurements were performed using an Autolab GSTAT128 N potentiostat/galvanostat (Metrohm-Autolab) controlled by NOVA 2.10 software. A Swagelok-type cell with 316 stainless steel electrodes of 1.0 cm in diameter, connected to the working electrode and the reference electrode to the two poles was used (Figure S3). EIS measurements were conducted at room temperature, with an alternating voltage amplitude of 10 mV and in a frequency range of 0.01 to 10^5 Hz. Equivalent circuits were obtained using the NOVA analysis program.

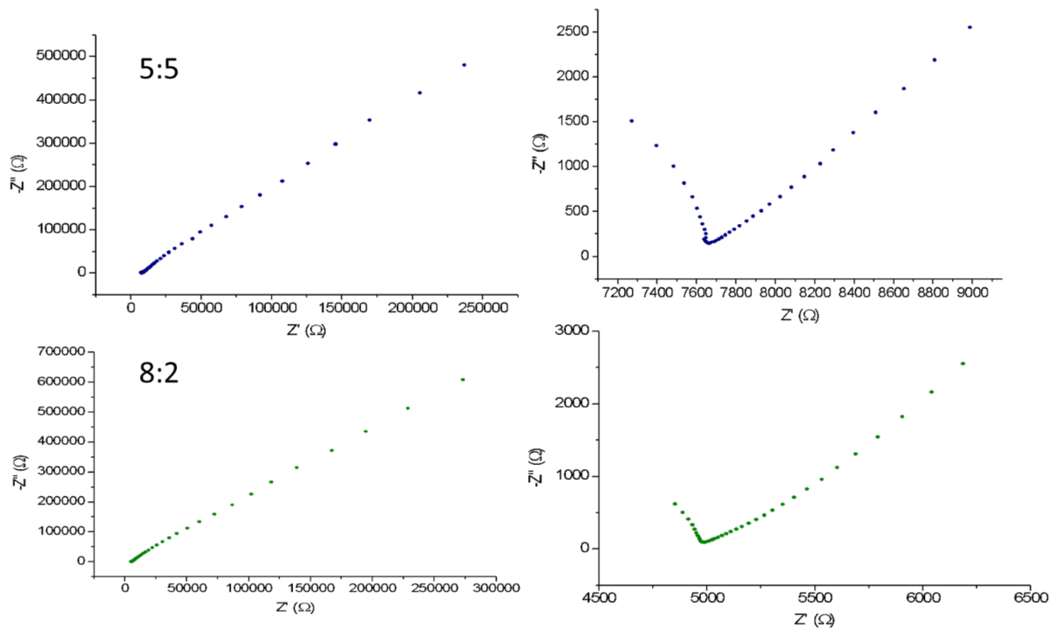

Figure S1 Nyquist plot obtained with 8:2 and 5:5 hydrogel

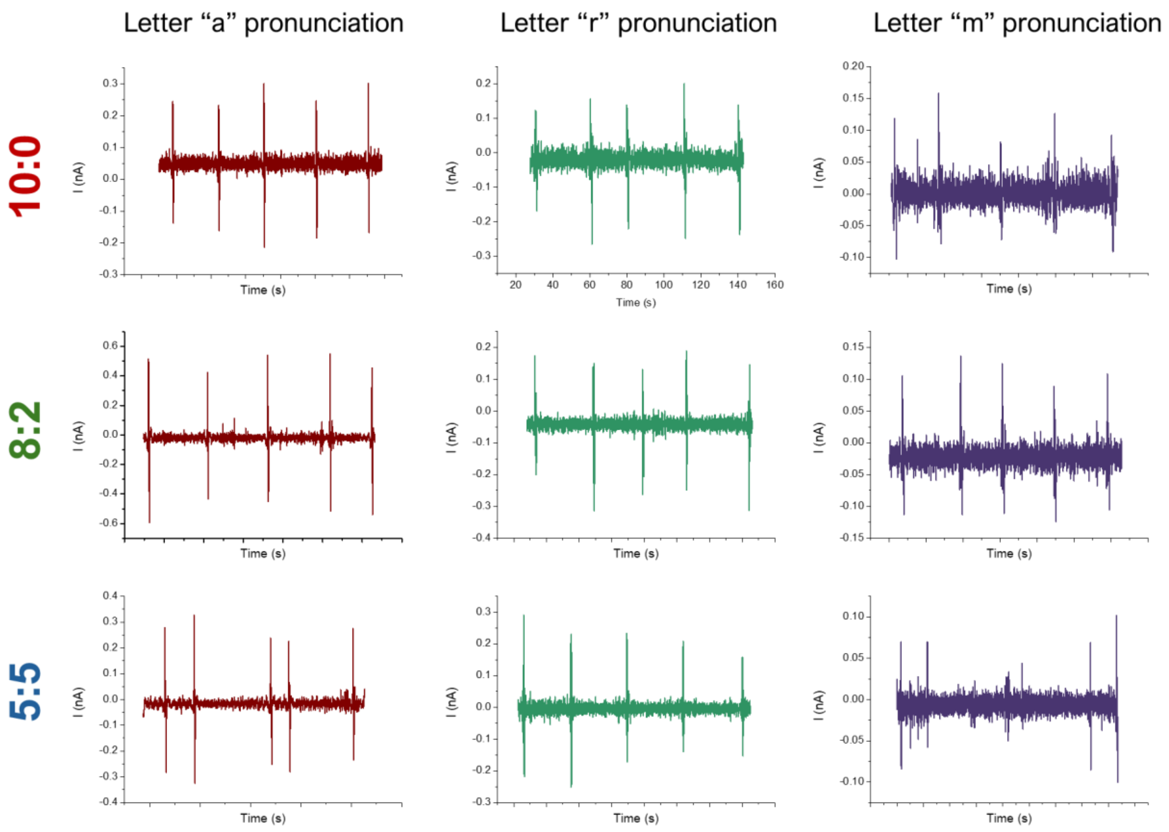

Figure S2 Comparison of current vs time signal for letters "a", "r" and "m", obtained with sensors 5:5, 8:2 and 10:0

Table S2. Recent advanced materials used in sensor applications

**Materials**	**Application**	**Mechanism**	**Ref**
Poly(vinyl alcohol)/Polyvinylidene fluoride hydrogel (PVA/PVDF Hydrogel)	Wearable pressure sensor	Triboelectric	This work
Carbon nanotubes + polyethyleneimine (CNT+PEI) and velvet or Polytetrafluoroethylene (PTFE)	Flexible pressure sensors	Triboelectric	[40][47]
Graphite coated with alumina and PTFE nanoparticles	Acoustic sensor	Triboelectric	[48]
Poly(vinyl alcohol)/Polyvinylidene fluoride fiber membrane (PVA/PVDF)	Energy harvesting and wearable sensors	Piezoelectric	[30] [49]
Polyacrylonitrile hydrogel incorporating ferroelectric poly(vinylidene fluoride) (PAN-PVDF)	Artificial Skin	Piezoelectric	[50]
PDMS fiber with rGO layer and Ag nanoparticle layer	Acoustic and strain sensors	Resistive	[51]
Triple-network (TN) cellulose nanofiber hydrogels	Wearable Electronics	Resistive	[52]
Noncracking vertically aligned gold nanowire (V-AuNW) films	Acoustic sensor	Resistive	[53]

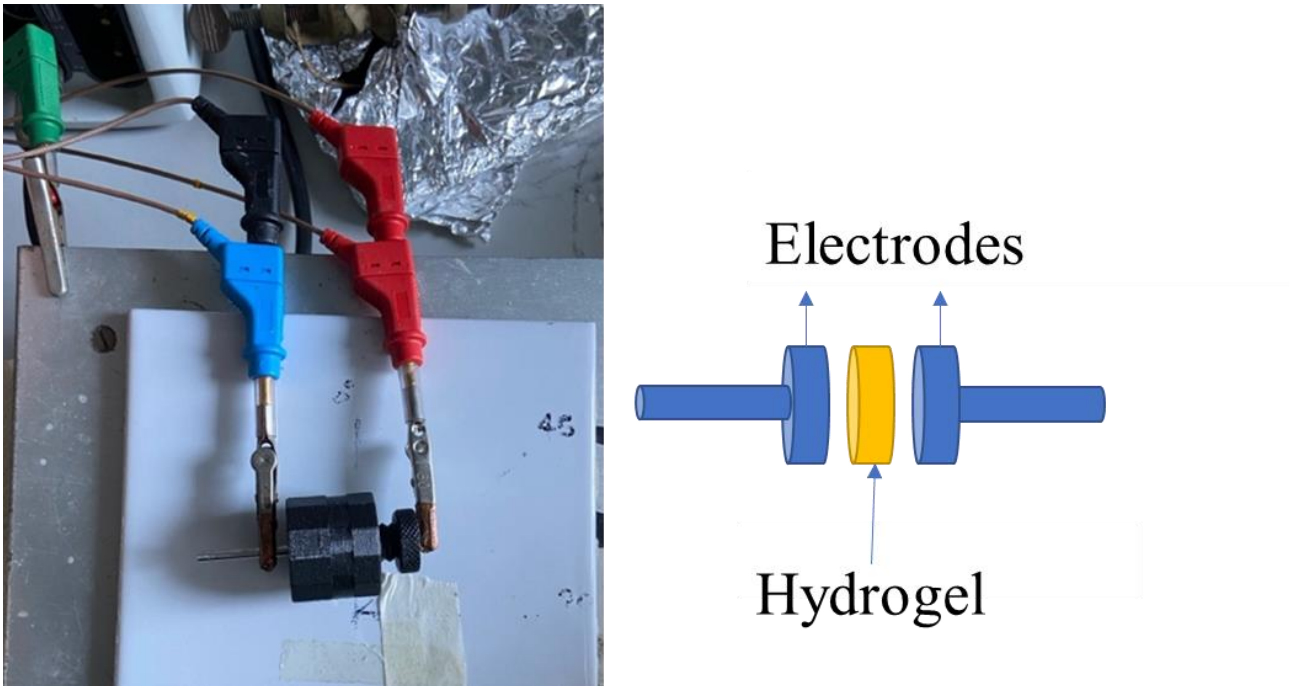

Figure S3. Experimental setup of the Swagelok cell (left) and scheme of the electrode and hydrogel assempbly (right)
